# Molecular mechanisms of KMT2C alterations in gastrointestinal cancers: enhancer network destabilization, lineage plasticity, and clinical translation

**DOI:** 10.3389/fimmu.2026.1799765

**Published:** 2026-05-28

**Authors:** Xintao Zeng, Pei Yang, Tao Wang, Ruizi Shi, Chuan Qin, Lun Zhang, Ting Jiang, Xi Chen, Hua Luo, Jihong Wei, Haiyan Hu, Decai Wang, Jianjun Wang

**Affiliations:** 1 Department of Hepatobiliary Surgery, Mianyang Central Hospital, School of Medicine, University of Electronic Science and Technology of China, Mianyang, China; 2 Department of Hepatobiliary Surgery, Union Hospital, Tongji Medical College, Huazhong University of Science and Technology, Wuhan, China; 3 Department of Urology, Mianyang Central Hospital, School of Medicine, University of Electronic Science and Technology of China, Mianyang, China

**Keywords:** DNA damage response, epigenetic biomarkers, epithelial–mesenchymal transition, H3K4me1, KMT2C, super-enhancers, tumor immune microenvironment

## Abstract

KMT2C (lysine methyltransferase 2C), also known as mixed-lineage leukemia 3 (MLL3), is a member of the KMT2 family of histone methyltransferases that catalyzes histone H3 lysine 4 monomethylation (H3K4me1), a hallmark of active enhancer elements. Operating within COMPASS-like complexes (Complex of Proteins Associated with Set1) and in association with the ASCOM coactivator complex (ASC-2–containing complex), KMT2C plays a central role in maintaining enhancer and super-enhancer integrity, thereby sustaining lineage-specific transcriptional programs. Across gastrointestinal malignancies, KMT2C is recurrently altered, predominantly through truncating loss-of-function variants, splice-disrupting events, and structural alterations that impair protein function. Importantly, the biological impact of KMT2C alteration is highly context dependent, shaped by mutation class, co-occurring genomic lesions, and tissue-specific transcriptional circuitry. Rather than inducing linear dysregulation of individual signaling pathways, KMT2C deficiency preferentially destabilizes enhancer and super-enhancer networks, leading to large-scale transcriptional rewiring. Disruption of enhancer modules that enforce cellular identity and homeostasis is frequently accompanied by activation of stress-adaptive and metabolic programs. Concurrently, defects in homologous recombination and replication-stress responses promote genomic instability, while attenuation of cell-cycle checkpoints and senescence barriers facilitates epithelial–mesenchymal transition, stem-like plasticity, and invasive or metastatic behavior. Beyond tumor-intrinsic effects, KMT2C dysfunction can reshape the tumor immune microenvironment through altered antigenic burden, inflammatory signaling, senescence-associated secretory programs, and dynamic stromal interactions, ultimately giving rise to heterogeneous therapeutic vulnerabilities. Clinically, KMT2C alteration has been linked to tumor mutational burden (TMB), microsatellite instability (MSI), immune infiltration patterns, and outcomes following immune checkpoint blockade (ICB). In parallel, KMT2C-associated DNA repair deficiencies provide a mechanistic basis for synthetic-lethal strategies involving poly(ADP-ribose) polymerase (PARP) inhibitors and inhibitors of ataxia telangiectasia and Rad3-related protein (ATR) or checkpoint kinase 1 (CHK1), including rational combinations with epigenetic therapies. In this review, we integrate evidence from hepatocellular carcinoma, pancreatic ductal adenocarcinoma, cholangiocarcinoma, colorectal cancer, gastric cancer, esophageal cancer, and gallbladder cancer within a unified framework that links KMT2C domain architecture to enhancer-network destabilization, phenotypic state transitions, and clinical manifestations. We further propose a functional evaluation paradigm that reframes discrete KMT2C variants as graded states of epigenetic deficiency, coupled with a closed-loop validation strategy integrating tissue-based profiling, liquid biopsy monitoring, and spatial multi-omics analyses.

## 1 Introduction

Gastrointestinal malignancies—including hepatocellular carcinoma, pancreatic ductal adenocarcinoma, cholangiocarcinoma, colorectal cancer, gastric cancer, esophageal cancer, and gallbladder cancer—are collectively characterized by pronounced heterogeneity, multistep evolutionary trajectories, and a strong dependence on the tumor microenvironment (1–8). In clinical practice, despite increasingly refined molecular classification systems and therapeutic strategies, substantial interpatient variability persists in tumor growth kinetics, recurrence patterns, and responses to systemic treatments, including targeted therapies and immunotherapies (9–12). Over the past decade, large-scale genomic sequencing efforts have identified numerous canonical driver mutations; however, these genetic alterations alone remain insufficient on their own to fully account for the remarkable phenotypic plasticity and adaptive capacity exhibited by gastrointestinal tumors during progression, metastasis, and therapeutic challenge (13–15). This discrepancy underscores the existence of higher-order regulatory mechanisms beyond the genome that continuously shape tumor evolutionary trajectories.

Among such mechanisms, epigenetic chromatin remodeling has emerged as a central interface linking genotype to phenotypic diversity and plasticity (16–22). In particular, enhancers—core cis-regulatory elements that determine cell type–specific transcriptional output—are highly responsive to chronic inflammation, fibrosis, metabolic stress, and therapeutic pressure, undergoing rapid and widespread reorganization under these conditions (23–26). Dynamic alterations in enhancer and super-enhancer landscapes not only rewire transcriptional regulatory networks but also destabilize established cell identity programs, thereby conferring substantial phenotypic plasticity and fitness advantages to tumor cells (27–31). Accumulating evidence indicates that enhancer reprogramming is not merely a passive byproduct of tumorigenesis, but rather represents a recurrent and shared epigenetic foundation underlying the transition of multiple gastrointestinal cancers from relatively differentiated states toward more aggressive and therapy-resistant phenotypes (32–36).

Within this regulatory framework, KMT2C (lysine methyltransferase 2C) functions as a key writer of enhancer-associated histone H3 lysine 4 monomethylation (H3K4me1) and plays a central role in maintaining cell type–specific enhancer identity and transcriptional network stability (37–41). Pan-cancer genomic analyses have revealed that KMT2C frequently harbors loss-of-function mutations across a wide spectrum of solid tumors and is associated with clinically relevant phenotypes—including invasive behavior, metastatic potential, recurrence risk, and response to immunotherapy—in multiple gastrointestinal malignancies (42–45). Unlike classical tumor suppressor genes, however, KMT2C alterations do not result in linear inactivation of a single signaling pathway. Instead, they are more appropriately conceptualized as systemic perturbations characterized by enhancer network imbalance, transcriptional program reconfiguration, and progressive drift in cellular states (31, 37–39, 44). The molecular and phenotypic consequences of KMT2C alterations are highly contingent upon mutation class and affected structural domains (44), co-occurring molecular contexts—such as alterations in TP53, KRAS, ARID1A, and DNA damage response (DDR)–related genes (43)—as well as lineage-specific transcription factor networks and microenvironmental selective pressures (39, 45).

Against this backdrop, this review systematically examines the molecular foundations of KMT2C function in transcriptional regulation from a structure–function coupling perspective, with particular emphasis on how KMT2C mutations contribute to gastrointestinal tumor initiation and progression through remodeling of enhancer and super-enhancer landscapes. Building on this framework, we further address the functional heterogeneity associated with distinct structural domains and mutation classes, aiming to elevate discrete “mutational events” into quantifiable, domain-informed, and clinically actionable states of functional deficiency. By integrating tissue lineage characteristics and molecular contexts across different gastrointestinal cancers, we also explore strategies for constructing biomarker systems linked to prognostic stratification and therapeutic response prediction, as well as the rational design of combination treatment approaches. Collectively, this review seeks to provide a testable conceptual framework that bridges enhancer reprogramming with clinical translation.

The distinctive contribution of this review is not simply to reiterate that KMT2C is an enhancer-associated epigenetic regulator, a concept that has been increasingly recognized in recent studies. Instead, we propose a clinically oriented framework in which KMT2C alterations are interpreted as graded functional deficiency states shaped by mutation class, affected structural domain, co-mutational background, tissue lineage, and therapeutic context. This framework differs from conventional mutation-centric or pathway-centric models by connecting domain-informed structural disruption with enhancer-network instability, transcriptional-state drift, and context-dependent therapeutic vulnerabilities. It also provides a practical rationale for integrating genomic annotation, transcriptional phenotyping, DNA damage response metrics, and immune microenvironmental features in future biomarker development.

## 2 Structural basis of KMT2C and its nodal role within enhancer regulatory networks

Understanding the biological functions of KMT2C in gastrointestinal malignancies requires situating this factor within the broader architecture of enhancer regulatory networks. Unlike epigenetic regulators that are traditionally characterized primarily by their enzymatic activity, KMT2C operates at the molecular level as a multifunctional integrator that coordinates catalytic activity, chromatin targeting, multiprotein complex assembly, and regulatory signal integration. In this regard, KMT2C is more appropriately conceptualized as an enhancer regulatory scaffold protein rather than a simple histone-modifying enzyme (37–41). Its domain composition and spatial organization jointly determine its occupancy across distinct enhancer repertoires, its cooperative interactions with transcription factors and co-regulators, and its capacity to stabilize higher-order super-enhancer architecture (28, 37, 38, 40, 41). Accordingly, dissecting how KMT2C contributes to the establishment and maintenance of enhancer networks from a structure–function coupling perspective provides a conceptual foundation for understanding how its alterations induce epigenetic imbalance and unleash lineage plasticity.

To avoid conceptual ambiguity, several terms are used consistently throughout this review. “KMT2C functional deficiency state” refers to the integrated biological consequence of KMT2C alteration, incorporating mutation class, affected structural domain, chromatin engagement, complex assembly, and downstream transcriptional output. “Enhancer destabilization” denotes disruption of enhancer or super-enhancer activity, hierarchy, or enhancer–promoter communication. “Transcriptional architecture instability” refers to higher-order reorganization of regulatory networks rather than isolated gene-level dysregulation. “Lineage plasticity” describes the increased capacity of tumor cells to shift between differentiated, dedifferentiated, mesenchymal-like, stem-like, or stress-adaptive states.

### 2.1 Domain architecture and functional stratification: the molecular basis of KMT2C as an enhancer regulatory scaffold

**Figure 1:**
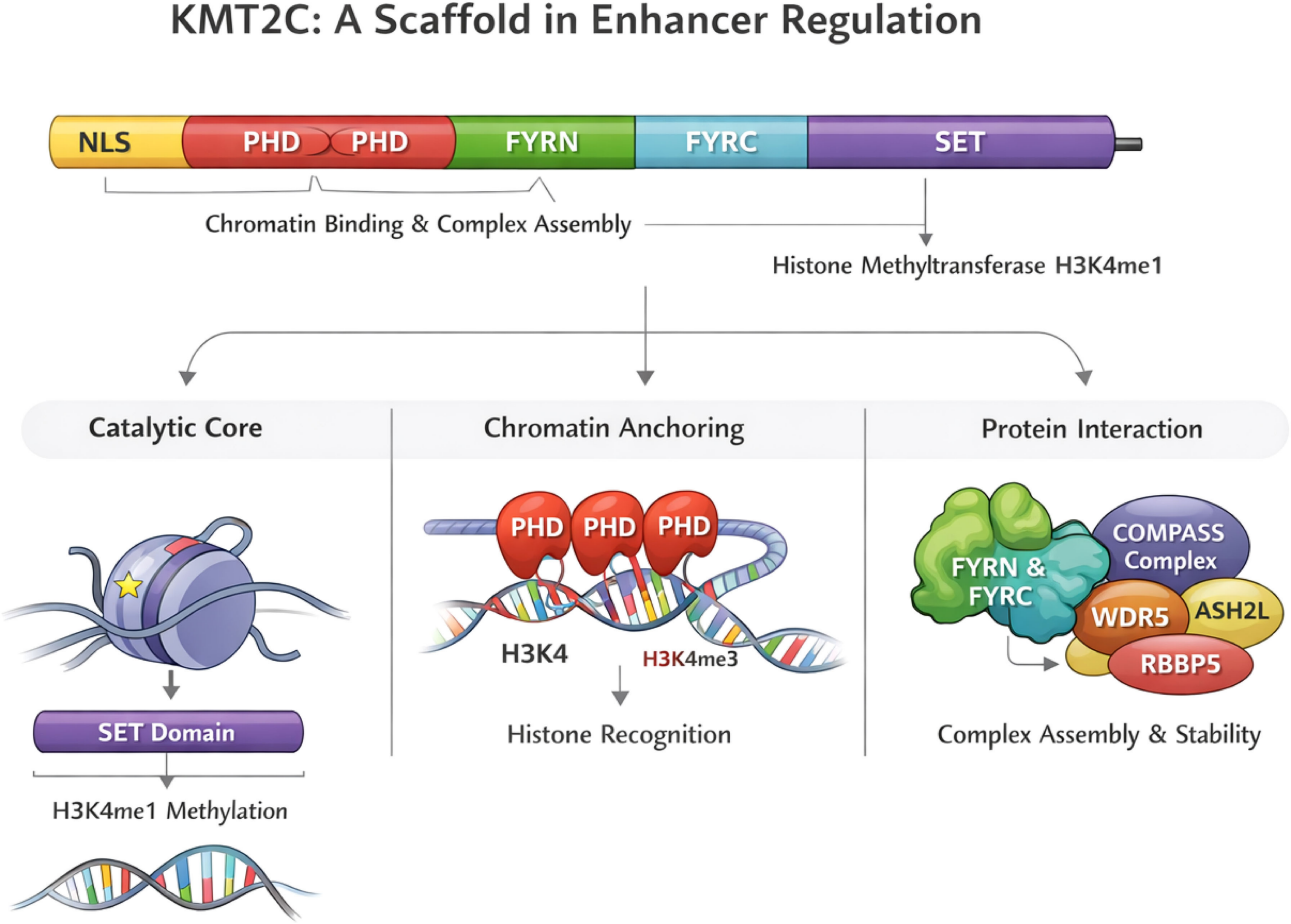
Domain-informed functional consequences of KMT2C alterations in enhancer regulation and transcriptional-state control. The figure summarizes the major structural domains of KMT2C/MLL3 and their functional relevance to enhancer-associated transcriptional regulation. The C-terminal SET domain mediates H3K4me1 deposition at enhancer regions and supports enhancer priming and maintenance. PHD finger motifs contribute to chromatin engagement and enhancer targeting, whereas the FYRN/FYRC regions participate in protein conformation, stability, and incorporation into COMPASS/ASCOM-related complexes. Accordingly, different classes of KMT2C alterations may produce distinct but partially overlapping functional consequences. Truncating or SET-disrupting mutations may impair catalytic activity, PHD-associated alterations may compromise chromatin targeting despite preserved catalytic capacity, and FYRN/FYRC perturbations may affect protein stability or complex assembly. These domain-level defects converge on enhancer and super-enhancer network destabilization, thereby contributing to transcriptional rewiring, lineage plasticity, altered DNA damage responses, immune microenvironment remodeling, and context-dependent therapeutic vulnerabilities.

The human KMT2C protein is exceptionally large and comprises multiple functional domains that cooperate across both structural and regulatory dimensions. Through this coordinated organization, KMT2C not only mediates histone-modifying enzymatic activity but also fulfills higher-order roles in chromatin localization, multiprotein complex assembly, and regulatory signal integration (**Figure 1**). From this perspective, KMT2C is best viewed as an enhancer regulatory scaffold protein rather than a standalone methyltransferase.

The C-terminal SET domain constitutes the catalytic core of KMT2C and is responsible for depositing histone H3 lysine 4 monomethylation (H3K4me1) at enhancer regions, a molecular event essential for the establishment and maintenance of enhancer identity (37, 41, 46, 47). Genomic analyses across pan-cancer and gastrointestinal tumor cohorts indicate that truncating or frameshift mutations resulting in loss of the SET domain are frequently associated with profound functional impairment, widespread transcriptional dysregulation, and unfavorable clinical outcomes (39, 43, 44, 46).

Beyond the catalytic domain, KMT2C contains multiple plant homeodomain (PHD) finger motifs that recognize specific histone modifications and contribute to chromatin anchoring. In this context, certain missense mutations that preserve SET domain integrity can nonetheless disrupt proper chromatin targeting of KMT2C, resulting in a functional state in which the catalytic machinery remains intact but is ineffectively delivered to relevant enhancer ensembles. Notably, the downstream phenotypic consequences of such mislocalization often substantially overlap with those observed in catalytically inactivating alterations, underscoring the importance of spatial chromatin engagement in KMT2C function (38, 40, 44, 46, 48).

Additional domains, including FYRN and FYRC, play critical roles in maintaining overall protein conformation and mediating protein–protein interactions. Structural perturbations affecting these regions can compromise KMT2C stability or impair its efficient incorporation into COMPASS/ASCOM-related complexes, thereby amplifying functional deficits at the systems level (40, 44, 46, 49).

Importantly, KMT2C rarely functions as an isolated enzyme within the cell. Instead, it acts as a core component of COMPASS/ASCOM-related complexes, cooperating with multiple subunits to determine its occupancy landscape across specific enhancer repertoires and its resultant transcriptional outputs (37, 38, 40, 49). Consequently, clinical and functional annotation of KMT2C alterations should move beyond a binary assessment of mutation presence or absence. Rather, priority should be given to evaluating affected structural domains, impacts on chromatin targeting or complex assembly, and integrated transcriptomic and epigenomic evidence in order to stratify the resulting states of functional deficiency.

Nevertheless, current evidence remains insufficient to assign every individual KMT2C variant to a precise biological output. Domain-level annotation should therefore be regarded as a practical stratification layer rather than a definitive functional classifier. Future studies integrating mutation mapping, enhancer profiling, transcriptomic output, and experimental validation will be required to determine whether specific mutation classes consistently correspond to distinct functional deficiency states.

### 2.2 A nodal role in enhancer and super-enhancer regulation: from permissive marking to stabilization of transcriptional architecture

At the epigenetic level, H3K4me1 deposited by KMT2C is widely recognized as a permissive or priming mark for enhancer elements. This modification establishes a molecular foundation that facilitates subsequent acquisition of H3K27 acetylation, recruitment of lineage-defining transcription factors and co-activators, and formation of long-range enhancer–promoter interactions (37, 41, 47, 50, 51). Under physiological conditions, these coordinated processes enable KMT2C to contribute to the construction and maintenance of cell type–specific enhancer networks, thereby ensuring the long-term stability of lineage-specific transcriptional programs.

When KMT2C function is compromised, the resulting consequences are rarely limited to stochastic inactivation of individual enhancers. Rather, KMT2C deficiency more commonly manifests as systemic remodeling of enhancer regulatory networks, characterized by redistribution of regulatory influence across distinct enhancer modules. On the one hand, enhancer activity supporting differentiation maintenance, tissue homeostasis, and tumor-suppressive programs is frequently attenuated, leading to repression of lineage identity genes and destabilization of differentiated cellular states (32, 37, 38, 44). On the other hand, enhancer modules associated with stress adaptation, metabolic reprogramming, migratory and invasive behavior, and therapeutic tolerance may acquire relative regulatory dominance through alternative control pathways, progressively reshaping the enhancer landscape in favor of oncogenic transcriptional programs (31–35).

At the level of super-enhancers, these imbalances are often further amplified. Given that super-enhancers typically orchestrate cell identity determinants or key oncogenic drivers, dysregulation of KMT2C can disrupt the composition, stability, and hierarchical organization of super-enhancer clusters, thereby exerting cascading effects on core transcriptional nodes (27–29, 52). Such perturbations not only accelerate transitions between discrete transcriptional states but also promote the continuous enrichment of phenotypic states that confer survival and fitness advantages during clonal selection and tumor evolution.

From a systems biology perspective, the central function of KMT2C thus extends far beyond the act of depositing a single histone modification. Rather, KMT2C serves to preserve the structural integrity and hierarchical organization of enhancer and super-enhancer networks (27, 28, 37, 38, 41). Functional deficiency of KMT2C therefore represents a destabilizing event at the level of transcriptional regulatory architecture, establishing an epigenetic substrate upon which lineage plasticity, cellular state drift, and tumor phenotypic evolution can subsequently unfold.

## 3 KMT2C shapes core tumor biological traits through enhancer reprogramming

From a mechanistic standpoint, functional impairment of KMT2C does not operate through selective perturbation of a single signaling pathway. Instead, it disrupts transcriptional homeostasis at a higher-order organizational level by reshaping enhancer and super-enhancer regulatory networks. This form of transcriptional architectural instability propagates along multiple biological axes and ultimately remodels the phenotypic landscape of tumor cells, influencing DNA damage responses, cell cycle and senescence control, cell fate stability, invasive and metastatic potential, and the organization of the tumor immune microenvironment. In essence, what is released by KMT2C deficiency is not the activity of a discrete pathway, but cellular state plasticity itself. Below, we discuss several interrelated dimensions of this process that also lend themselves to translational interrogation.

### 3.1 Impaired DNA damage response and genomic instability: enhancer destabilization and a hypomorphic DDR

Accumulating evidence indicates that KMT2C contributes to the transcriptional homeostasis of multiple genes involved in DNA damage response (DDR) pathways and replication stress surveillance (31, 38, 43, 44, 49). Functional deficiency of KMT2C compromises genome maintenance through at least two complementary mechanisms. First, systemic remodeling of enhancer landscapes leads to coordinated downregulation of genes involved in homologous recombination, replication fork protection, and cell cycle checkpoint control (37, 38, 44). Second, alterations in chromatin accessibility and local chromatin architecture may further impair the efficient recruitment and assembly of repair factors at sites of DNA damage, thereby weakening DDR execution at the chromatin interface (53–55).

Notably, this impairment rarely manifests as a classical, fully penetrant DDR deficiency. Instead, it more closely resembles a hypomorphic DDR state, in which core repair pathways remain partially functional under basal conditions, yet become insufficient under sustained replication stress or exogenous genotoxic challenge. In this context, KMT2C-deficient cells frequently exhibit delayed repair kinetics, replication fork instability, and progressive accumulation of DNA double-strand breaks (44, 53, 56). Over time, this state accelerates the accrual of chromosomal aberrations and mutational burden, thereby continuously fueling genetic diversification during tumor clonal evolution (44, 57).

From a translational perspective, this hypomorphic DDR state carries dual implications. On the one hand, it constitutes an intrinsic driver of tumor evolution and intratumoral heterogeneity. On the other hand, in specific molecular contexts, it may create exploitable therapeutic vulnerabilities, potentially rendering KMT2C-deficient tumors more sensitive to synthetic lethal strategies involving poly(ADP-ribose) polymerase inhibitors or inhibitors of ataxia telangiectasia and Rad3-related protein (ATR) and checkpoint kinase 1 (CHK1) (44, 58–61). Accordingly, the coupling between KMT2C functional status and homologous recombination deficiency scores, replication stress metrics, and functional DDR readouts warrants systematic evaluation across multi-cancer cohorts.

It should be emphasized, however, that current evidence linking KMT2C deficiency to DDR impairment is derived primarily from pan-cancer and tumor-specific cohort analyses, mechanistic studies in cellular and animal models, and retrospective associations with treatment response. While these data demonstrate strong cross-level concordance, prospective clinical studies that stratify patients based on KMT2C functional deficiency and directly assess benefit from DDR-targeted therapies remain limited. Thus, this vulnerability should currently be regarded as a translational hypothesis supported by robust mechanistic and cohort-level evidence, rather than an established clinical determinant.

### 3.2 Cell cycle deregulation, senescence escape, and stress adaptation: from transcriptional brake release to survival advantage

Under homeostatic conditions, KMT2C contributes to maintaining enhancer activity across transcriptional modules that enforce cell cycle restraint and senescence-associated programs, including the p53–p21 axis and related cyclin-dependent kinase inhibitor networks (e.g., p27), thereby constraining aberrant proliferation and stabilizing cell fate (37, 38, 58, 59). When KMT2C function is compromised, this transcriptional braking system is frequently weakened through enhancer-network remodeling, manifesting as attenuation of cell cycle checkpoint control and blunted activation of senescence-associated programs.

Concurrently, enhancer reprogramming can reinforce transcriptional programs that support stress survival, including antioxidant defense, hypoxia-responsive signaling, and metabolic remodeling under nutrient limitation (31, 32, 34, 35). These changes may enable tumor cells not only to bypass proliferative constraints but also to sustain survival and growth within hostile tumor ecosystems shaped by chronic inflammation, fibrosis, or hypoperfusion, thereby conferring a selective fitness advantage *in vivo* (62–64).

Accordingly, the phenotype associated with KMT2C deficiency is not simply accelerated proliferation. Rather, it is better conceptualized as a composite state in which weakened cell cycle restraint is coupled with enhanced stress adaptability. This state may be captured by multidimensional signatures integrating senescence-associated transcriptional modules, oxidative stress responses, and metabolic adaptation programs, and is consistent with reported associations between KMT2C deficiency, therapeutic resistance, and relapse risk in multiple contexts (31, 34, 35, 44, 59).

At present, evidence supporting a role for KMT2C in regulating cell cycle restraint, senescence, and stress adaptation derives mainly from epigenomic/transcriptomic association analyses and functional studies *in vitro* and *in vivo*. While these data implicate KMT2C as an important regulator of cellular state transitions, direct causal links between this mechanistic axis and specific clinical outcomes—such as recurrence patterns or treatment resistance—remain to be established through prospective cohorts stratified by KMT2C functional deficiency and supported by orthogonal functional readouts.

### 3.3 Epithelial–mesenchymal transition, invasion, metastasis, and tumor stemness: epigenetic release of cellular state plasticity

At the level of cell fate control, one of the most characteristic consequences of KMT2C deficiency is attenuation of the transcriptional constraints that maintain differentiated cellular states, thereby unleashing cellular state plasticity (31, 32, 37). Phenotypically, this has been frequently associated with activation of epithelial–mesenchymal transition (EMT)–associated transcription factor networks, enhanced migratory and invasive capacity, and sustained expression of stemness-associated transcriptional programs (32, 65).

Mechanistically, these changes are not driven by a single linear signaling axis. Rather, they emerge from global reorganization of enhancer network hierarchy, in which enhancer modules sustaining epithelial differentiation and tissue identity progressively weaken, whereas regulatory modules associated with migration, inflammatory signaling, and stemness gain relative regulatory dominance. As this redistribution of enhancer influence accumulates over time, tumor cells become increasingly capable of transitioning between functional states, facilitating adaptation during local invasion, dissemination, and ectopic colonization (31–34).

In addition, KMT2C deficiency can engage in positive feedback loops with stromal remodeling and inflammatory signaling, contributing to the establishment of a permissive pre-metastatic niche and promoting secondary reprogramming of tumor cell states at metastatic sites. These reciprocal interactions may enhance the efficiency of distant organ colonization and sustained outgrowth following dissemination (33, 34, 66). Collectively, these observations suggest that KMT2C-associated EMT and stemness phenotypes are better conceptualized as dynamic, partial, or hybrid states rather than irreversible terminal differentiation programs (67, 68).

It is important to note that current evidence linking KMT2C deficiency to partial EMT and stem-like states is largely derived from multi-cancer transcriptomic analyses and experimental models, with correlative associations observed in selected clinical cohorts. Whether this relationship constitutes an independent determinant of invasion, metastasis, or clinical outcome beyond co-mutation backgrounds remains to be rigorously tested in prospective studies that adequately control for confounding variables.

### 3.4 Remodeling of the tumor immune microenvironment and context-dependent responses to immunotherapy

The impact of KMT2C alterations on the tumor immune microenvironment is characterized by pronounced duality and strong context dependence. On the one hand, consistent with its association with increased genomic instability, a subset of KMT2C-deficient tumors may exhibit elevated neoantigen burden and, in specific molecular contexts, increased tumor mutational burden (TMB) or microsatellite instability (MSI)–associated features. Together, these alterations may provide a potentially permissive immunogenic substrate for immune checkpoint blockade (44, 45, 69).

On the other hand, transcriptional reprogramming induced by KMT2C deficiency has been frequently observed to co-occur with epithelial–mesenchymal transition and reinforcement of senescence-associated secretory phenotypes. These processes can upregulate multiple immunosuppressive pathways, including cytokine and chemokine signaling, immune checkpoint ligand expression, and cues that promote recruitment of immunosuppressive cellular populations such as regulatory T cells, myeloid-derived suppressor cells, and immunosuppressive macrophage subsets. Collectively, these effects may effectively counterbalance—or even obscure—any intrinsic immunogenic advantage conferred by increased mutational burden (70–73).

Consequently, the predictive significance of KMT2C status for immunotherapy response is not unidirectional but is instead highly contingent upon the surrounding molecular and microenvironmental context. In clinical evaluation, KMT2C status should therefore be interpreted in conjunction with programmed death-ligand 1 (PD-L1) expression, tumor mutational burden, microsatellite instability status, and immune infiltration profiles, rather than being considered in isolation.

At present, associations between KMT2C status, immune microenvironmental features, and response to immunotherapy are derived primarily from retrospective cohort analyses and multi-omic correlation studies, with notable inconsistencies across tumor types and treatment settings. Accordingly, KMT2C should currently be regarded as a candidate state marker of immune niche remodeling rather than an established independent predictor of immunotherapy benefit, pending validation in prospective immunotherapy cohorts stratified by functional deficiency states.

### 3.5 Boundary conditions and apparent paradoxes of KMT2C function: why enhancer destabilization is not uniformly oncogenic

Although KMT2C deficiency is frequently associated with enhancer destabilization, lineage plasticity, and aggressive tumor behavior, the available evidence does not support a uniformly oncogenic interpretation across all biological contexts. Several apparently conflicting observations have been reported, including associations between reduced KMT2C expression and cell-cycle inhibition, growth restriction, or more favorable prognosis in selected settings. These discrepancies likely reflect differences in how KMT2C status is defined and measured, particularly the distinction between quantitative downregulation of KMT2C expression and qualitative structural disruption of the KMT2C protein. They may also be influenced by tumor-type-specific transcriptional circuitry, co-mutational background, treatment exposure, and other dataset-specific confounders. Therefore, a balanced interpretation requires separating expression-based observations from mutation-based functional impairment.

First, reduced KMT2C expression is not equivalent to structural loss-of-function mutations. Multiple studies have shown that, in certain cancer types or experimental models, downregulation of KMT2C expression can correlate with cell cycle inhibition or even more favorable prognosis (53, 74, 75). In contrast, truncating, frameshift, or domain-disrupting mutations more consistently correspond to destabilization of enhancer networks and release of lineage plasticity (38). These observations indicate that quantitative changes in gene expression and qualitative structural disruption exert fundamentally different—and in some contexts opposing—biological effects, with only the latter reliably representing a transcriptional architectural destabilization event.

Second, the directionality of KMT2C-associated effects is highly dependent on the co-mutation landscape. In molecular backgrounds characterized by TP53 loss, KRAS activation, ARID1A deficiency, or pre-existing impairment of DNA damage response pathways, KMT2C deficiency is more likely to be amplified into a coupled trajectory of plasticity release and genomic instability (53, 74). Conversely, in relatively genomically stable settings, or in contexts in which dominant oncogenic drivers have already fixed lineage identity, KMT2C deficiency may instead provoke cell cycle restraint or excessive survival stress, manifesting as apparent growth-restrictive effects (75). Thus, KMT2C functions less as a binary switch and more as a system-level modulator or amplifier, with its output determined by the pre-existing regulatory landscape (38, 74, 76).

Third, tissue lineage–specific transcriptional networks critically determine how enhancer destabilization is translated into phenotypic output. Distinct gastrointestinal organs are governed by fundamentally different combinations of lineage-determining transcription factors and enhancer topologies (28, 29). In some tissues, loosening of enhancer architecture preferentially manifests as epithelial–mesenchymal transition, stemness maintenance, and migratory advantage (77), whereas in others it may primarily lead to replication stress accumulation, metabolic imbalance, or even survival disadvantage (78). This lineage-dependent dimension offers a mechanistic explanation for why similar KMT2C alterations can be associated with divergent—or even opposing—clinical implications across cancer types.

Finally, therapeutic pressure itself represents a powerful selective force in tumor evolution, reshaping clonal architecture and the cellular state space (79). During untreated natural disease progression, KMT2C deficiency may predominantly confer invasive and plasticity-related advantages. Under immunotherapy, radiotherapy, chemotherapy, or targeted therapy, however, the same functional state may diverge along distinct evolutionary trajectories: on the one hand, forming an epigenetically plastic platform for therapy tolerance (80); on the other, exposing exploitable immune or DNA damage response vulnerabilities due to accompanying genomic instability or inflammatory signaling (81, 82). Accordingly, the clinical relevance of KMT2C alterations is, to a large extent, treatment-context dependent.

Taken together, KMT2C should not be simplistically categorized as either a tumor suppressor or an oncogene. A more mechanistically coherent view is that KMT2C functions as a system-level homeostatic factor that stabilizes enhancer architecture and lineage-specific transcriptional programs. The biological and clinical consequences of its deficiency emerge from the combined effects of mutation structure, co-mutation landscape, tissue lineage context, and therapeutic selection pressure. This framework provides a unifying rationale for the heterogeneous and sometimes paradoxical clinical associations of KMT2C alterations discussed in the following cancer-specific sections.

## 4 Biological consequences and clinical implications of KMT2C mutation–driven lineage reprogramming in gastrointestinal cancers

This section examines how KMT2C-associated enhancer destabilization is translated into cancer type-specific biological and clinical phenotypes across gastrointestinal malignancies. Rather than reiterating a single universal mechanism, we focus on how tissue lineage, co-mutational background, microenvironmental context, and therapeutic pressure shape the consequences of KMT2C deficiency in each tumor type.

Because the amount and quality of evidence vary substantially across gastrointestinal cancer types, the following cancer-specific discussion distinguishes direct evidence from mechanistic inference where appropriate. For relatively well-studied tumors such as hepatocellular carcinoma, colorectal cancer, and gastric cancer, KMT2C alterations have been linked to clinical phenotypes and, in some cases, experimentally supported biological mechanisms. In contrast, for less extensively studied entities such as gallbladder cancer and certain esophageal cancer subtypes, current evidence remains more limited, and the proposed mechanisms should be interpreted as hypothesis-generating rather than definitively established.

### 4.1 Hepatocellular carcinoma

In hepatocellular carcinoma, KMT2C is most coherently conceptualized as an enhancer gatekeeper of hepatocyte lineage stability. Unlike many epithelial malignancies, the dominant biological tension in HCC arises from the chronic convergence of inflammation, metabolic stress, and regenerative pressure. Within this context, even modest relaxation of enhancer homeostasis can be amplified into pronounced lineage drift and dedifferentiation (77, 83). Cohort-level analyses generally indicate that KMT2C alterations in HCC are enriched for truncating, frameshift, or structural loss-of-function events and are associated with increased invasiveness, elevated recurrence risk, and unfavorable clinical outcomes (84). Importantly, these associations are less consistent with a simple mutation–proliferation paradigm than with a shift from lineage constraint toward a permissive state of epigenetic plasticity.

Mechanistically, the impact of KMT2C deficiency in HCC can be delineated along two principal and interacting axes. First, attenuation of enhancer modules responsible for maintaining hepatocyte identity leads to silencing of differentiation and homeostatic programs, thereby facilitating dedifferentiated, stem-like, or multilineage transcriptional states (85–87). Second, stress-response and metabolic adaptation programs are reinforced, enabling tumor cells to better tolerate hypoxia, nutrient fluctuation, and immune-mediated pressure (84, 88). Within this landscape, MYC-associated transcriptional circuitry frequently functions as a context-dependent amplifier: under physiological conditions, KMT2C partially constrains MYC-driven proliferative and metabolic outputs, whereas loss of this constraint allows growth-promoting modules to dominate the enhancer hierarchy and shape an adaptively favorable transcriptional configuration (87, 89, 90). Concurrent destabilization of cell-cycle checkpoint modules, including those linked to CDKN2A, may further bias tumor cells toward plasticity-favored survival under high replication stress rather than durable growth arrest, thereby accelerating evolutionary dynamics (84, 87, 91).

From a translational perspective, the clinical utility of KMT2C in HCC should be embedded within a functional deficiency framework rather than assessed solely by mutation presence or absence. Pragmatic strategies include: (i) constructing composite risk models that integrate mutation class and domain disruption with transcriptional signatures of lineage loosening—such as partial epithelial–mesenchymal transition or stemness-associated programs—and indices of DNA damage response or replication stress; (ii) prioritizing rational combination regimens that suppress plasticity-driving programs (for example, MYC- or metabolism-linked axes) while concurrently exploiting DNA damage response vulnerabilities or enhancing immune efficacy; and (iii) prospectively defining which KMT2C functional deficiency states reliably map to actionable DNA damage response or immune sensitivity, and validating their predictive robustness for recurrence risk and treatment benefit.

### 4.2 Pancreatic ductal adenocarcinoma

Pancreatic ductal adenocarcinoma provides a particularly informative setting in which to interrogate the context dependency of KMT2C function. A densely fibrotic and hypovascular microenvironment imposes strong selective pressure for stress adaptation and metabolic remodeling, while immunosuppressive myeloid populations and stromal barriers markedly restrict immune penetration (92–95). Within this ecological niche, KMT2C deficiency is more likely to reinforce enhancer programs governing stress tolerance and survival, enabling tumor cells to sustain proliferation under hostile conditions. Several studies have linked KMT2C alterations to metabolic pathway activation, shifts in immune composition, and adverse clinical outcomes—findings that are broadly consistent with a model in which enhancer architectural reorganization rewires both tumor-intrinsic transcriptional programs and extrinsic ecological interactions (96–98).

Critically, PDAC requires a clear distinction between reduced KMT2C expression and bona fide structural loss-of-function mutations (97, 99). Multiple reports have described associations between low KMT2C expression and cell-cycle suppression or even more favorable outcomes, whereas genetic depletion or domain-disrupting mutations in specific experimental systems can induce growth arrest or stress intolerance in a context-dependent manner (97). These observations underscore a bidirectional, state-dependent relationship in which the biological output of KMT2C perturbation is constrained by the co-mutation spectrum and lineage-specific transcriptional circuitry. Consequently, the central translational question in PDAC is not mutation status per se, but the nature of the functional deficit and the transcriptional state it engenders.

Of particular interest are KRAS–wild-type PDAC subsets, in which KMT2C alterations have been reported to be enriched (100–102). Given the broader therapeutic latitude of this subgroup, a stable linkage between KMT2C functional deficiency and a defined enhancer reprogramming–driven stress-adaptation state could provide a basis for refined patient stratification. Therapeutically, when functional and microenvironmental stratification is rigorously applied, DNA damage response–centered strategies may be prioritized in settings characterized by homologous recombination deficiency or replication stress vulnerability, whereas epigenetic–immunotherapy combination approaches may be explored in contexts showing partial immune niche loosening or inflammatory activation.

### 4.3 Cholangiocarcinoma

The pathobiological substrate of cholangiocarcinoma is shaped by prolonged cholestasis, chronic inflammation, and extensive fibrosis—conditions under which epigenetic homeostasis may be particularly vulnerable to destabilization (103, 104). Relatively elevated frequencies of KMT2C mutations have been reported in certain high-risk subsets, including younger patients with aggressive disease courses, suggesting a potential role for KMT2C deficiency in steering tumors toward unfavorable evolutionary trajectories (105). Rather than equating KMT2C deficiency with direct oncogenic activation, a more coherent interpretation is that it acts as a trigger that accelerates entry into a plastic, adaptive transcriptional state. Once enhancer order is relaxed, programs linked to epithelial–mesenchymal transition, stromal interaction, and immune evasion can be co-activated in a modular and context-dependent manner, yielding resistant phenotypes that are further reinforced by fibrotic barriers and inflammatory signaling within the tumor microenvironment (104, 106).

Clinically, the value of KMT2C in cholangiocarcinoma lies less in its use as a standalone biomarker than in its incorporation into composite predictive frameworks. Embedding KMT2C status within next-generation sequencing panels and circulating tumor DNA monitoring—together with spatial fibrosis phenotypes, immune infiltration profiles, and plasticity-associated transcriptional signatures—may help identify patient subgroups potentially amenable to immuno-epigenetic combination strategies (107–110). Given the strongly immunosuppressive baseline and prominent stromal constraints characteristic of cholangiocarcinoma, immunosensitization approaches are likely to require concurrent targeting of stromal accessibility and myeloid suppression in order to translate biological signals into meaningful *in vivo* therapeutic benefit.

### 4.4 Colorectal cancer

In colorectal cancer, KMT2C is among the most frequently altered chromatin regulators (111). However, its clinical relevance depends less on mutation frequency per se than on whether it provides incremental stratification beyond established classification frameworks, particularly those anchored in microsatellite instability, tumor mutational burden, and immune infiltration. From an enhancer reprogramming perspective, KMT2C deficiency may alter the relative weighting of Wnt-driven lineage programs, inflammatory response modules, and cell-cycle control networks, thereby increasing reversible transitions between differentiated and dedifferentiated transcriptional states. Such state instability may promote intratumoral heterogeneity expansion, with downstream implications for recurrence risk and metastatic potential (33, 112). Rather than forcing KMT2C into a single pathway-centric framework, a state-based risk modeling approach that integrates dedifferentiation programs, epithelial–mesenchymal transition–related modules, and indices of DNA damage response or replication stress is more conceptually aligned with its systems-level role.

In the immunotherapy setting, reported correlations between KMT2C deficiency, elevated tumor mutational burden or microsatellite instability–associated phenotypes, and altered immune landscapes suggest potential utility as an auxiliary stratification factor rather than a standalone predictor (44, 45, 69). In microsatellite instability–high or tumor mutational burden–high colorectal cancer, KMT2C loss-of-function may further refine inflamed transcriptional backbones within otherwise immunogenic tumors. Conversely, in microsatellite-stable disease, if KMT2C deficiency predominantly reflects plasticity release coupled with immunosuppressive remodeling, epigenetic priming strategies combined with immunotherapy may be conceptually rational. In such settings, validation using spatially resolved profiling will be critical to distinguish superficial inflammatory signaling from immune exclusion imposed by stromal architecture.

### 4.5 Gastric cancer

Gastric cancer provides one of the most illustrative clinical contexts in which to observe the context dependency of KMT2C function. Multiple studies have associated KMT2C mutation or reduced expression with disease progression, recurrence risk, and metastatic dissemination, while distinct alteration patterns observed across primary and metastatic sites suggest a role in clonal selection and microenvironmental adaptation (113). Experimental evidence further supports an association between KMT2C deficiency and activation of epithelial–mesenchymal transition–related programs, leading to enhanced migratory and invasive capacity (114). Notably, this phenotype is more accurately described as a partial or hybrid EMT state that preserves proliferative potential while conferring migratory fitness, consistent with heterogeneous dissemination routes and therapy-resistant trajectories observed clinically (115).

Conversely, in subsets of gastric cancer treated with immune checkpoint inhibitors, KMT2C mutations have been associated with increased CD8^+^ T-cell infiltration and improved therapeutic responses, suggesting that under specific molecular and microenvironmental contexts, KMT2C deficiency may coincide with heightened immunogenicity or a pre-existing inflammatory baseline (116–119). These observations need not be contradictory. Rather, they can be unified by a state-based model in which enhancer imbalance–driven transcriptional drift promotes invasive adaptation, while—in the presence of genomic instability or inflammatory signaling—simultaneously creating conditions permissive for immune engagement. The central clinical challenge is therefore to distinguish tumors dominated by an immune-inflamed reprogramming axis from those governed by an epithelial–mesenchymal transition/senescence-associated secretory phenotype–myeloid suppression–stromal barrier axis.

Accordingly, evaluation of KMT2C as a predictive biomarker in advanced gastric cancer is likely to require an integrated framework that combines tissue-based next-generation sequencing, longitudinal circulating tumor DNA monitoring, and immune or spatial phenotyping, rather than reliance on any single metric.

### 4.6 Esophageal cancer (ESCC/EAC)

In esophageal cancer, the relationship between KMT2C and the senescence barrier is most coherently interpreted through its role in enhancer-mediated transcriptional control of cell-cycle restraint programs. MLL3/KMT2C is an integral component of enhancer regulatory machinery that stabilizes lineage- and cell-state–specific transcriptional programs, including modules governing proliferation arrest and cellular stress responses (37, 40). Disruption of enhancer-mediated transcriptional control has been shown to compromise the robustness of p53–p21–centered and related cyclin-dependent kinase inhibitor–dependent growth arrest programs, thereby facilitating escape from therapy-induced senescence into plastic survival states (52, 120).

In esophageal squamous cell carcinoma (ESCC), therapy-induced senescence constitutes a critical barrier to durable tumor control following chemoradiotherapy. Clinical and experimental evidence indicates that failure to establish or maintain stable senescence programs enables residual tumor cells to re-enter the cell cycle and drive disease recurrence (121, 122). In this therapeutic context, loss of KMT2C-mediated enhancer stabilization is likely to weaken the transcriptional integrity of senescence-associated programs and instead bias stressed tumor cells toward a plasticity-favored survival trajectory characterized by enhanced adaptive capacity.

Accordingly, translational studies in esophageal cancer should prioritize treatment-anchored cohorts to determine whether KMT2C functional deficiency correlates with impaired senescence-associated transcriptional modules, altered stress-response programs, and distinct recurrence patterns. Such analyses could further inform the rational design of combination strategies integrating DNA damage response–targeted or epigenetic therapies with radiotherapy and immunotherapy, particularly in subsets marked by high replication stress or rewritable cell-state landscapes.

### 4.7 Gallbladder cancer

Given its rarity and pronounced heterogeneity, gallbladder cancer remains underexplored with respect to KMT2C alterations (123, 124). Rather than extrapolating detailed mechanistic models from limited and fragmented evidence, a more appropriate positioning is to view GBC as a real-world testing ground for functional deficiency–based stratification. Initial efforts should prioritize comprehensive characterization of KMT2C mutation spectra and co-mutation patterns in multicenter, real-world cohorts, and relate these features to treatment exposure and clinical outcomes. Subsequently, stratification of patient-derived organoid or patient-derived xenograft models by mutation class and domain disruption—integrated with enhancer activity profiling and systematic drug sensitivity assays—could determine whether KMT2C deficiency reproducibly maps onto defined plasticity states or exploitable therapeutic vulnerabilities. Success of such a closed-loop framework would not only inform gallbladder cancer management but also critically test the generalizability and boundary conditions of the KMT2C-driven enhancer reprogramming model across biliary tract malignancies.

Across gastrointestinal cancers, the shared epigenetic substrate of KMT2C deficiency lies in relaxation of enhancer and super-enhancer regulatory order, leading to reduced lineage stability and accelerated transcriptional state drift. The resulting clinical manifestations, however, are shaped by tissue-intrinsic transcriptional backbones, cooperative mutational contexts, and microenvironmental as well as therapeutic selection pressures. Consequently, KMT2C should not be reduced to a single predictive biomarker. Instead, it is best conceptualized as a nodal regulator within a functional deficiency state framework, integrated with enhancer activity metrics, lineage state indicators, DNA damage response vulnerability, and immune ecological features. Such an approach supports the rational design of combination strategies aimed at exploiting context-specific vulnerabilities while constraining maladaptive plasticity, and provides a unifying translational roadmap across gastrointestinal malignancies.

## 5 Future perspectives and directions for clinical translation

### 5.1 From “mutation events” to “functional deficiency states”: establishing an actionable translational framework

It should be emphasized that the proposed state-based stratification model is intended as a translational framework for prospective validation rather than an established clinical decision-making standard. Its potential advantage over mutation-only biomarkers lies in its ability to integrate upstream genetic disruption with downstream transcriptional, DNA repair, and immune phenotypes, thereby better capturing the functional consequences of KMT2C deficiency.

To render the concept of a functional deficiency state both actionable and reproducible across centers, a staged translational strategy is preferable to an idealized, “all-at-once” multi-omics approach. In view of current clinical feasibility and prevailing research workflows, we outline a two-tier operational framework for evaluating KMT2C functional deficiency: a minimal, clinically implementable framework and an enhanced, research-grade framework.

The minimal clinically implementable framework is designed to capture the dominant biological consequences of KMT2C deficiency without materially increasing diagnostic burden. At its core, it integrates three complementary dimensions. First, mutation class and domain-level annotation derived from routine next-generation sequencing—such as truncating alterations, loss of the catalytic SET domain, or missense variants affecting PHD-associated regions—can provide initial genetic stratification. Second, RNA-based signatures reflecting enhancer reprogramming and lineage destabilization, obtained from transcriptomic data or targeted expression profiling—encompassing dedifferentiation, partial epithelial–mesenchymal transition, stemness, or stress-adaptation modules—can quantify downstream cellular-state outputs. Third, functional indicators of genome maintenance vulnerability, approximated by homologous recombination deficiency scores, replication stress–associated gene sets, or validated surrogate markers, can offer an orthogonal readout of DNA damage response capacity and replication stress burden. Joint consideration of these three dimensions yields a pragmatic and scalable approximation of KMT2C functional deficiency within existing clinical testing infrastructures, enabling prospective association with recurrence risk, metastatic propensity, and treatment sensitivity.

Building on this foundation, the enhanced research-grade framework incorporates higher-resolution epigenetic and spatial information to resolve intrastate heterogeneity and microenvironmental constraints. In representative cohorts or exploratory studies, integrating enhancer-associated histone modification profiles, chromatin accessibility measurements, and spatial transcriptomic or proteomic data can enable fine-grained mapping of enhancer and super-enhancer reorganization, spatial partitioning of lineage states within tumors, and their coupling to immune infiltration and stromal architecture. Importantly, this tier is not intended for routine clinical deployment; rather, it serves to inform feature selection, weighting, and threshold calibration for the minimal framework on mechanistic grounds.

This “use-first, refine-later” strategy avoids the common pitfall whereby state-based models appear valid only under comprehensive multi-omics conditions. Instead, it allows KMT2C-related assessment to mature iteratively and empirically into a reproducible and generalizable stratification tool that can be deployed in real-world settings to support patient selection, therapeutic prioritization, and rational combination-therapy design.

### 5.2 Biomarker development pathways: closed-loop validation from tissue to liquid biopsy and spatial multi-omics

Because KMT2C-associated phenotypes exhibit pronounced spatiotemporal heterogeneity and evolve dynamically under clonal selection, biomarker development cannot be reliably confined to any single analytical layer. A closed-loop validation strategy that triangulates tissue-based profiling, liquid biopsy, and spatial multi-omics is therefore required to derive robust and clinically portable markers.

At the tissue level, systematic characterization of mutation class, truncation position, domain involvement, and co-mutation landscapes using next-generation sequencing provides a baseline framework for functional stratification. At the liquid biopsy level, longitudinal tracking of circulating tumor DNA enables real-time capture of clonal and transcriptional state dynamics, facilitating early detection of lineage-state shifts that accompany treatment response, disease progression, or emergent resistance, and allowing temporal alignment with imaging and clinical endpoints. Finally, in representative tumor samples, spatial multi-omics—including spatial transcriptomics, spatial epigenomic profiling, or high-dimensional spatial proteomics—can resolve how enhancer remodeling, immune infiltration, and stromal interactions are organized *in situ* under KMT2C-deficient conditions. This spatial layer is particularly well suited to addressing a central translational question: why tumors assigned to similar KMT2C functional deficiency states can nevertheless display divergent therapeutic responses across distinct microenvironmental contexts.

The value of this pathway does not lie in the accumulation of technologies, but in the rigor of implementation. Prospective cohort design, harmonized sample processing, and interpretable statistical modeling are essential to ensure standardization and scalability, enabling functional deficiency states to transition from a research construct into a predictive tool reproducible across centers. Without such rigor, even mechanistically compelling biomarkers are likely to fail at the point of clinical adoption.

### 5.3 Therapeutic frontiers: epigenetic–DNA damage response–immune combination strategies guided by state-based stratification

The therapeutic relevance of KMT2C deficiency derives from its ability to drive enhancer reprogramming and release lineage plasticity. Rather than conferring selective advantage through a single linear signaling pathway, KMT2C-deficient tumors enter a high-plasticity transcriptional state that is permissive to state transitions and adaptive remodeling. Accordingly, future therapeutic development is best organized around two complementary design principles—exploiting induced vulnerabilities and constraining maladaptive plasticity—with state-based functional stratification as a prerequisite.

First, synthetic lethal strategies targeting vulnerabilities in DNA damage response and replication stress pathways warrant systematic prioritization. In KMT2C-deficient tumors that exhibit homologous recombination impairment or defective replication stress responses, rational evaluation of poly(ADP-ribose) polymerase inhibitors and inhibitors of ataxia telangiectasia and Rad3-related protein (ATR) or checkpoint kinase 1 (CHK1)—administered sequentially or in combination—should be guided by mechanistic evidence, with integration alongside radiotherapy or chemotherapy where appropriate (58, 59,125). A central challenge is to define which specific KMT2C functional deficiency states reliably translate into actionable DNA repair vulnerabilities, rather than relying on mutation presence alone as a sufficient selection criterion.

Second, immuno-sensitization strategies that target enhancer reprogramming and immune niche remodeling represent a complementary therapeutic axis. In immunologically “cold” gastrointestinal tumors—such as subsets of microsatellite-stable gastric and colorectal cancers, as well as prototypically immunosuppressive pancreatic ductal adenocarcinoma and cholangiocarcinoma—epigenetic modulators may, in principle, be used to reconfigure enhancer landscapes and antigen presentation programs, with the goal of improving T-cell infiltration and effector function prior to immune checkpoint blockade. Crucially, immuno-sensitization is not achieved through simple drug addition. Its success is constrained by the underlying microenvironmental substrate; in settings dominated by epithelial–mesenchymal transition–associated secretory programs, suppressive myeloid enrichment, or dense fibrotic barriers, stromal interactions and inflammatory circuitry must be treated as first-order design variables rather than secondary modifiers.

In sum, the objective of KMT2C-informed therapeutic research is not to identify a single “KMT2C-targeted drug,” but to leverage functional stratification to pinpoint tractable vulnerabilities within highly plastic tumors and to deploy combination strategies that simultaneously exploit these liabilities while constraining maladaptive plasticity. Only through stringent biomarker-guided patient selection and rigorous mechanistic validation can combination regimens avoid unnecessary toxicity in non-target populations and fully realize the translational promise of moving from enhancer reprogramming to clinical intervention.

## 6 Conclusion

In summary, KMT2C deficiency should not be viewed as an isolated epigenetic mutation event, but rather as a transcriptional architecture–level perturbation centered on destabilization of enhancer and super-enhancer networks, leading to erosion of lineage stability and acceleration of cellular state transitions. Depending on tissue lineage, co-mutation context, and therapeutic environment, this state can be translated into distinct clinical phenotypes, including enhanced invasiveness, adaptive therapeutic resistance, or exploitable vulnerabilities in immune surveillance and DNA damage response pathways. Consequently, the translational value of KMT2C should move beyond a binary, gene-centric view of mutation presence toward a quantifiable framework of functional deficiency states. Looking forward, therapeutic strategies that combine suppression of plasticity-driving programs with exploitation of concomitant DNA damage response or immune vulnerabilities may provide a rational direction for KMT2C-informed intervention. However, their clinical value will depend on prospective validation using state-based, rather than mutation-only, stratification approaches.
